# The Insurance Act and Party Politics

**Published:** 1912-05-04

**Authors:** 


INDEXED

### The Hospital

The Workers' Newspaper of Administrative Medicine and Institutional Life, Administration, National Insurance and Health.

No. 1347, Vol. LII. SATURDAY, MAY 4, 1912. PRICE ONE PENNY.

### THE INSURANCE ACT AND PARTY POLITICS.

We offer no apology for reverting once more to this topic, on which so much has already been said, because in our deliberate judgment the Act whose operation is to begin in July will affect the community more nearly than any single piece of legislation in recent years. It is not only the hospitals, great and small; the doctors, of all degrees and gradations; the institutional workers, from the chairman of the board to the last recruited ward-maid; but the people themselves, who in their own daily lives will find the conditions of existence modified by the operation of National Insurance. How far those modifications of social economy will be beneficial to those concerned by them depends largely on such modifications of the provisions of the Act, as enacted, as can be brought about in the next twelve weeks.

The Act is capable of proving one of the great blessings which Parliament has conferred upon the democracy; but only if it is substantially altered from its present form in many respects. Otherwise it stands a very fair chance indeed of proving unworkable and unendurable to those whom it so nearly concerns—the employers and the employed. The Commissioners have large powers, extraordinary powers indeed, of affecting such improvements as the House of Commons, owing to the exigencies of party politics, was given no time to devise last year. And it is fairly clear that, in so far as they have the power, they will reconstruct the faulty parts of the half-finished edifice handed over to their care. But there are some defects which are fundamental, and, indeed, the very foundations of the Act; and these, as we have pointed out already on previous occasions lately, only Parliament itself can remedy. At the moment the really pressing problem confronting Messrs. George and Masterman is the firm stand made by the doctors. The practitioners are proving so unexpectedly stiffnecked that they threaten to wreck the whole scheme from the outset. Threats and vituperation have failed to move them; and the representatives who are now negotiating on their behalf are probably less pliant than the politician-surgeon who proved so complaisant to deal with last summer. It is interesting to note the next move in the game, now apparently being prepared.

The difficulty is very largely a financial one, for the wage-limit demand could be conceded by the Insurance Commissioners. The latter body can do much within the four corners of this elastic Act, but they cannot manufacture money, and therefore they cannot concede the terms for which the British Medical Association is holding out. Ministerialist "lobby gossip," therefore, is crediting the Chancellor of the Exchequer with the intention of increasing the contribution of the State, beyond the twopence per insured person per week already provided, to meet the deficiency and satisfy the doctors. To find the necessary money this year, it is said, a portion of the realised surplus of last year may be voted. Now this rumour, widely as it is credited, is, of course, merely a rumour; but it will not be astonishing if it turns out to be a feeler which has been inspired from influential quarters. The suggestion to postpone until next year the commencement of the operations of the Act has been steadfastly resisted by the Government, who are perfectly well aware by this time of the fixed determination animating the medical profession. So it may not unreasonably be inferred that they hope to circumvent this obstacle somehow or other. The threat to suspend medical benefit we have always regarded as bluff. To increase the contributions of either the employer or the employee would be to enhance the serious unpopularity of the Act, which has already cost more by-elections than the Coalition can afford. The only remaining cow to be squeezed is the taxpayer; so squeezed he is not at all unlikely to be.

With the financial ethics of this proposal we are not concerned, beyond this: that such a raid, for the purpose of propping up a tottering Act, upon money diverted from the Sinking Fund will be most fiercely resented and contested by the Opposition, and will certainly be unpalatable to a good many of the Ministerialists. It would strain party loyalty to such an extent that it might, conceivably, even lead to a disruptive crisis, though this is a contingency which is certainly remote. Even were the move safely affected, the money would have to be provided in the Estimates in future years. Assuming, however, that the doctors were thus bought off, there yet remain many more matters to be adjusted before any smooth and satisfactory working of the Act can be expected. Further, the hospitals, the agricultural labourers, and half-a-dozen other malcontent sections of the community, will all profit by the example set them, and press their demands with renewed persistency. What will happen? He would be a bold man that would undertake to foretell.

